# Resistance Mechanisms Influencing Oncolytic Virotherapy, a Systematic Analysis

**DOI:** 10.3390/vaccines9101166

**Published:** 2021-10-12

**Authors:** Darshak K. Bhatt, Roger Chammas, Toos Daemen

**Affiliations:** 1 University Medical Center Groningen, Department of Medical Microbiology and Infection Prevention, University of Groningen, 9713 AV Groningen, The Netherlands; d.bhatt@umcg.nl; 2 Center for Translational Research in Oncology, Instituto do Câncer do Hospital das Clínicas, Faculdade de Medicina, Universidade de São Paulo, São Paulo 01246-000, Brazil; rchammas@usp.br

**Keywords:** oncolytic virotherapy, therapeutic resistance, stromal cells, cancer cells, resistance mechanisms

## Abstract

Resistance to therapy is a frequently observed phenomenon in the treatment of cancer, and as with other cancer therapeutics, therapies based on oncolytic viruses also face the challenges of resistance, such as humoral and cellular antiviral responses, and tumor-associated interferon-mediated resistance. In order to identify additional mechanisms of resistance that may contribute to therapeutic failure, we developed a systematic search strategy for studies published in PubMed. We analyzed 6143 articles on oncolytic virotherapy and found that approximately 8% of these articles use resistance terms in the abstract and/or title. Of these 439 articles, 87 were original research. Most of the findings reported pertain to resistance mediated by tumor-cell-dependent interferon signaling. Yet, mechanisms such as epigenetic modifications, hypoxia-mediated inhibition, APOBEC-mediated resistance, virus entry barriers, and spatiotemporal restriction to viral spread, although not frequently assessed, were demonstrated to play a major role in resistance. Similarly, our results suggest that the stromal compartment consisting of, but not limited to, myeloid cells, fibroblasts, and epithelial cells requires more study in relation to therapy resistance using oncolytic viruses. Thus, our findings emphasize the need to assess the stromal compartment and to identify novel mechanisms that play an important role in conferring resistance to oncolytic virotherapy.

## 1. Introduction

Therapeutic resistance has been studied as a mechanism that allows the target to escape and evolve against the treatment, for example in the context of antibiotic resistance by pathogenic bacteria and resistance by tumors towards radiotherapy, chemotherapy, or immunotherapy. In the early phase of research assessing cancer resistance, most of the resistance mechanisms were attributed to be innate to tumors [1]. However, recent findings have revealed that therapeutic resistance may also arise via evolutionary mechanisms. A detailed understanding of such mechanisms is therefore required to improve therapeutic outcomes.

Recently, oncolytic virotherapy has been considered a promising biotherapy, where native or genetically engineered viruses are utilized to infect and kill cancer cells [2,3]. Moreover, it has been demonstrated that such oncolytic viruses, in addition to tumor killing, can also promote local and systemic antitumor immune responses and thus contribute to improved therapeutic outcomes [4]. Nevertheless, oncolytic virotherapy is not equally effective in all patients and cancer types, thus suggesting the possibility of therapeutic resistance. Although cellular and humoral antiviral immune responses have been well documented in the context of infectious virology, there have been fewer studies that explore resistance mechanisms in terms of cancer and virotherapy. To this end, we conducted a systematic review with the aim to provide a comprehensive overview of the resistance mechanisms associated with cancer and stromal cells and viruses employed for oncolytic virotherapy. We considered that resistance would not only be a result of an antiviral immune response, but also be a response mediated by tumor cells and stromal cells present in the tumor microenvironment. Therefore, through this systematic review, we hope to shed light on some of the overlooked players and mechanisms of resistance that undermine the therapeutic efficacy of oncolytic viruses.

## 2. Materials and Methods

Protocol: We used the Preferred Reporting Items for Systematic Review and Meta-Analysis Protocol (PRISMA-P) statement as a guide to formulate the research question and process articles for analysis.

**Figure 1:**
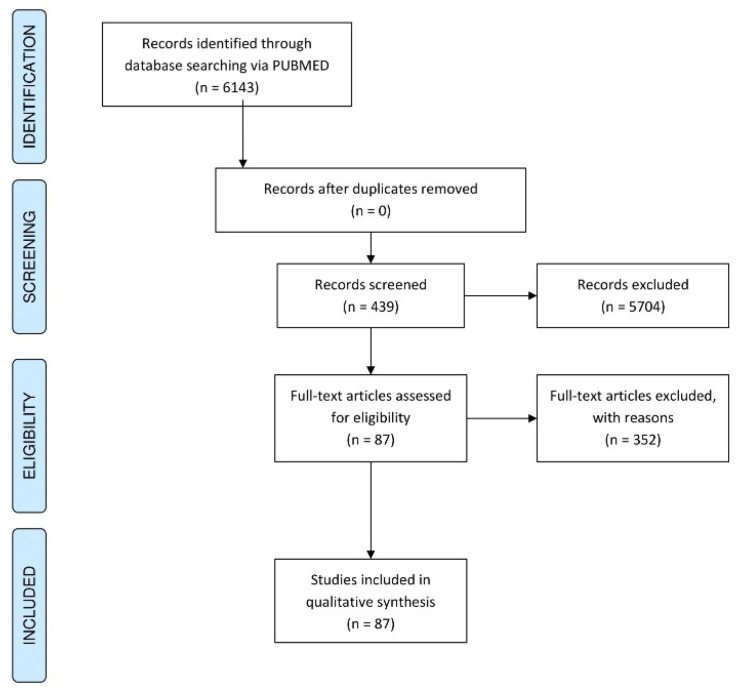
PRISMA flow diagram indicating the steps of article screening for a systematic analysis.

Eligibility criteria: We included all studies published to date that had the terms “oncolytic” and “resistance” in their abstract and/or title. The search strategy was thus defined as “resistance”[tiab] AND “oncolytic”[tiab] for exploring articles on PubMed. Only original research, in the form of full-text English publications, was included for analysis. Abstracts, letters, reviews, and commentaries were excluded (indicated in Figure 1).

Search strategy and article screening: A systematic search strategy was developed for PubMed. Titles that met our eligibility criteria were identified and processed for study selection. Title and abstract screenings were performed. Subsequently, a detailed analysis of the selected 87 articles was performed. Any conflicts were resolved through discussion. All data and results are provided in Supplementary Table S1 and are intended to serve as a resource and inspiration for future studies in the field of oncolytic virotherapy. Figures were made using the Orange Data Mining software [5] and the RawGraphs platform [6]. The graphical abstract was made using Biorender.

## 3. Results

A systematic search on PubMed performed on articles retrieved on 31 January 2021 found that 6143 articles contained “oncolytic”-related terms in the abstract and/or title, while only 439 of these articles (7.2%) contained both the terms “resistance” and “oncolytic” in the abstract or title.

**Figure 2:**
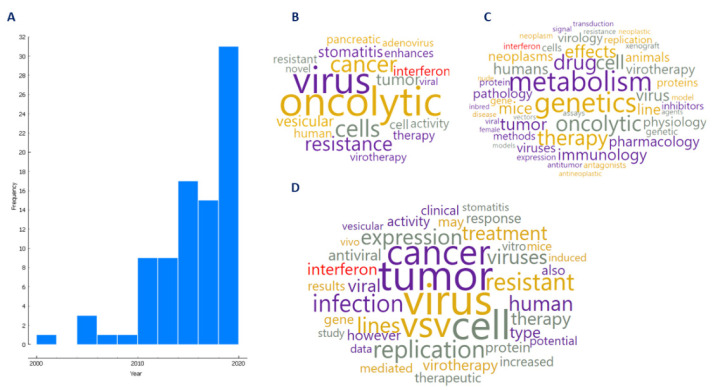
Literature review in the context of resistance to oncolytic viruses. (**A**) Number of original research articles (a total of 87) that studied resistance to virotherapy in the context of cancer from 2000 to 2020. (**B**) Frequently used terms in article titles, (**C**) MESH heading terms, and (**D**) abstracts.

A detailed screening of the 439 articles revealed 87 original research articles (1.5%) that studied resistance to virotherapy in the context of cancer. Regarding these 87 articles, Figure 2A shows that from the year 2000 to 2020, there has been an increase in the number of original research articles pertaining to resistance in oncolytic virotherapy. The most frequent terms used in the article title (Figure 2B) MeSH heading terms (Figure 2C) and terms from the abstract (Figure 2D) are an indication of the diversity of the concepts that relate to this subject. Of note, “interferon” (marked in red in Figure 2B–D) was found to be one of the most frequently retrieved terms from titles, abstracts, and MeSH headings, thus indicating that it might be a well-studied resistance mechanism in the literature.

**Figure 3:**
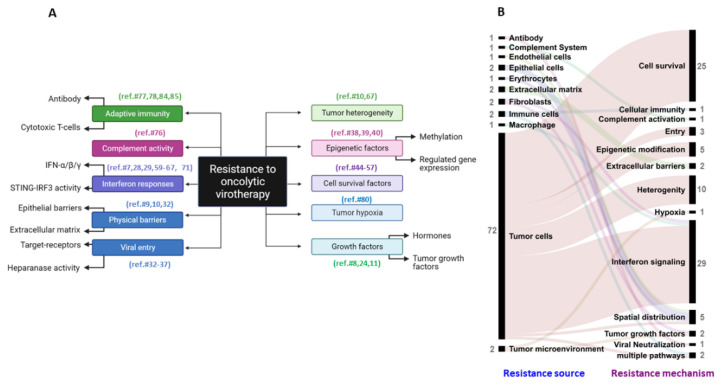
Resistance mechanisms undermining the efficacy of oncolytic viruses. (**A**) Types of resistance mechanisms and responsible molecular factors studied in reference to oncolytic viruses. The reference number of articles corresponding to the respective resistance mechanisms is provided in brackets. (**B**) Frequency of studies focusing on a particular resistance source and corresponding mechanism. Each link indicates an article. The number of articles studying a particular resistance source and resistance mechanism is indicated next to the label in grey.

These 87 articles were published in more than 40 journals by various authors. Seventeen viral vector types (natural or genetically modified) were studied in relation to resistance developed in twenty-two cancer types represented by hundreds of cell lines and preclinical animal models. This analysis also indicated an increasing interest in understanding the role of therapeutic resistance towards oncolytic viruses since the last decade (since 2010). Resistance mechanisms could be classified based on molecular features along with cellular mechanisms and cell-type-specific mechanisms (indicated in Figure 3A,B). Through this approach, we found that there has been a selective focus on assessing interferon (IFN)-mediated resistance by tumor cells (twenty-seven studies), with only two studies reporting on the role of fibroblast-mediated resistance [7,8] and three other studies assessing the role of epithelial cells and endothelial cells as physical barriers in regulating the spatiotemporal spread of the virus in tumors [9,10,11] (Figure 3B). Interestingly, two studies assessed the role of tumor-associated myeloid cells (TAMs) in conferring resistance to oncolytic virotherapy; however, only one of them elaborated on macrophage-mediated interferon signaling as a mechanism of resistance [12].

**Figure 4:**
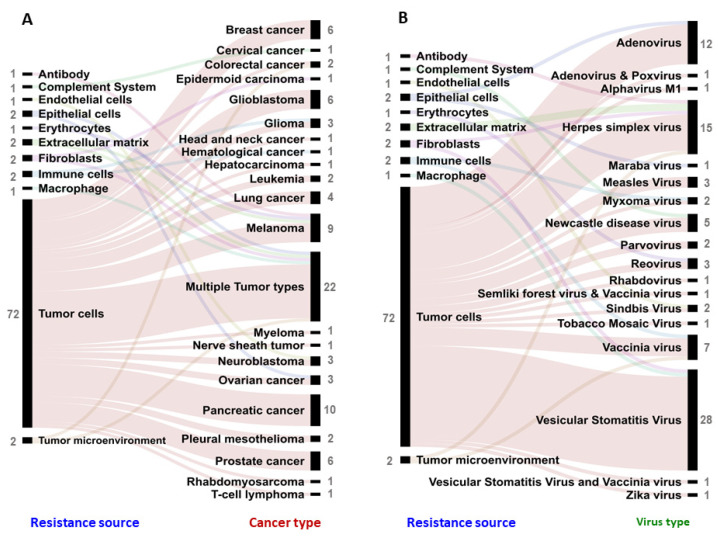
Resistance to oncolytic virotherapy exhibited by different sources or cell types present in the tumor microenvironment. Resistance source assessed in research articles in the context of (**A**) different cancers and (**B**) different virotherapy platforms. Each link indicates an article. The number of articles studying a particular resistance source, cancer type, and virus type is indicated next to the label in grey.

Resistance to virotherapy has been studied in different cancer types. Pancreatic cancer (10 studies), melanoma (9 studies), prostate cancer (6 studies), breast cancer, and glioblastoma have been the most studied individual cancer types in the context of resistance, where tumor-cell-mediated resistance mechanisms are frequently explored (72 studies) (Figure 4A). Yet, resistance mechanism studies also often include cancer cell lines belonging to different cancer types (Figure 4A) in a single study (22 studies), thus enabling high-throughput screening of various pathways associated with resistance [8,10,12,13,14,15,16,17,18,19,20,21,22,23,24,25,26,27,28,29,30,31].

A wide range of virus types have been described to exhibit decreased therapeutic efficacy due to resistance mechanisms (Figure 4B). The most frequently described viral vectors in this context were vesicular stomatitis virus (VSV, 29 studies), which is an enveloped RNA virus, herpes simplex virus (HSV, 15 studies,) an enveloped DNA virus, and adenovirus (AdV, 12 studies), a nonenveloped DNA virus (Figure 4B). Interestingly, more research with VSV, HSV, and AdV has led researchers to uncover a wide range of resistance mechanisms that stem from tumor cell heterogeneity (10 studies), epigenetic modifications (5 studies), hypoxia (1 study), blocking viral entry (3 study), and association of novel cell survival genes (25 studies) in the process of either preventing productive infection and/or subsequent oncolysis (Figure 4B).

**Figure 5:**
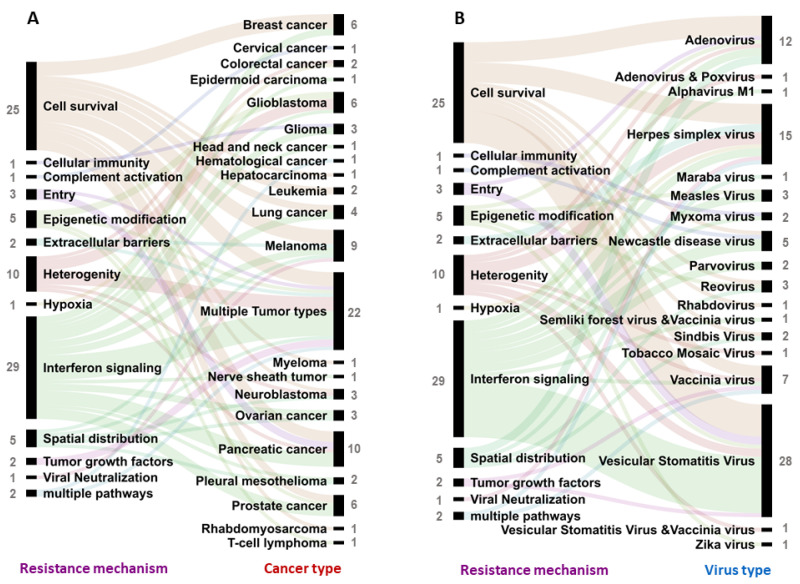
Assessing resistance towards oncolytic viruses in various cancers. Resistance to oncolytic virotherapy exhibited by different mechanisms studied in various articles in the context of (**A**) different cancer types and (**B**) towards different virotherapy platforms. Each link indicates an article. The number of articles studying a particular cancer type, virus type, and resistance mechanism is indicated next to the label in grey.

A common mechanism explored in various cancer types is the activation of interferon-mediated antiviral signals (29 studies) and the activation of cell-survival molecular mechanisms that protect tumor cells against viral infection and infection-induced oncolysis (25 studies) (Figure 5A). It is moreover important to note that resistance mechanisms associated with tumor growth factors (2 studies), physical barriers (2 studies), and preventing viral spread in tumor tissue (5 studies) and mechanisms that involve systemic responses of a multidimensional nature are frequently associated with the tumor microenvironment and not a particular cell type (Figure 5A and Figure 3B). Once more, interferon-mediated resistance and cell survival mechanisms have been studied more often across a wide range of virus types (Figure 5B). It is important to note here that other resistance mechanisms may or may not play a role in reducing the efficacy of different oncolytic viruses; however, the absence of such a study prevents one from reaching such a conclusion.

## 4. Discussion

This systematic analysis was based on 87 articles retrieved from published literature describing current practices in assessing resistance to oncolytic virotherapy. The aim of many of these articles was to explore which cellular pathways are associated with resistance and, if possible, to regulate these pathways and overcome resistance for better therapeutic outcomes. Of note, the most frequently studied resistance mechanisms, irrespective of virotherapy type, were based on cellular signaling post-virus infection, often focusing on interferon responses (29 studies) mediated by tumor (27 studies) and stromal cells (2 studies). Tumor-cell-mediated resistance mechanisms were explored more (72 studies) in comparison to stromal-cell-based mechanisms (8 studies), where pancreatic cancer (10 studies), melanoma (9 studies), prostate cancer (6 studies), breast cancer (6 studies), and glioblastoma (6 studies) were the cancer types most explored. The present results indicate the complexity of resistance phenomena and the challenge in targeting such mechanisms while designing virotherapy. Thus, for a comprehensive understanding of these mechanisms retrieved from the systematic analysis, we discuss in detail below how molecular processes regulated by various cell types present in the tumor microenvironment can impact the efficacy of virotherapy. Accordingly, we classify the resistance mechanisms studied in these articles as: tumor-cell-mediated responses, stromal cell responses, antiviral immune responses, and systemic responses.

Tumor cell-mediated responses: Tumor cells are the primary targets of oncolytic virotherapy. Therefore, resistance developed by, or innate in, tumor cells against viral activity dictates the clinical outcome of the therapy. This might explain why most of the studies (72 studies) retrieved from the systematic analysis had a focus on tumor-cell-mediated resistance. Resistance to viral infection and replication by tumor cells can be achieved at four distinct stages of (1) virus binding and entry, (2) viral transcription–translation, (3) cell survival, and (4) cell signaling. Here, it is important to note that a decrease in the oncolytic potential of a virus could be a result of resistance mechanisms that directly prevent virus replication and the translation of viral proteins or be due to virus inactivation in the extracellular space and disposal due to systemic processes. To assess various mechanisms associated with resistance to viral gene therapy, a study by Song et al. analyzed and identified changes in tumor gene expression post-virus infection. They found that these differentially expressed genes were involved in regulating the above-mentioned four stages by altering cell morphology, the expression of cell surface proteins affecting virus binding and entry, and altering cell development, movement, growth, and proliferation and cell-to-cell signaling and interaction [16].

Virus binding and entry: Virus entry depends on multiple factors, including the presence of host cell receptors for virus binding and the presence of neutralizing antibodies that block uptake, but also the presence of proteases in the extracellular matrix, which can either promote or prevent virus binding. Few studies (<5 studies) retrieved from the systematic analysis, were designed to study virus entry as a mechanism of resistance of oncolytic virotherapy.

For pleural mesothelioma, downregulation of heparanase enzymes in the tumor microenvironment has been described to result in the formation of a dense extracellular matrix, preventing deep tumor infiltration of viruses and attachment to target cells [32]. Pancreatic ductal adenocarcinoma cell lines have been described to have a low expression of the low-density lipoprotein receptor, the receptor for vesicular stomatitis virus, thus conferring a resistant phenotype to the tumor cells [33]. Moreover, epidermal growth factor receptor-targeted HSV has shown to be sensitivity to key neutralizing antibodies that pre-dominantly target glycoprotein D as a viral attachment protein [34].

Additionally, protein kinase B (AKT) activity and virus-induced activation of the pathway regulated by the mammalian target of rapamycin (AKT/mTOR axis) is linked to resistance in various cancers. Choi et al. described how the endogenous activity of AKT and related mutations promoted virus entry of Orthopoxvirus in triple-negative breast cancer (cells) [35]. Tong and colleagues showed that the AKT/mTOR axis can be regulated by blocking phosphoinositide 3-kinase (PI3K) activation, thus sensitizing multiple cancer cell lines in vitro for viral infection [36]. Rapamycin was observed to reverse mTOR-mediated tumor cell resistance against herpes simplex virotherapy, indicating that molecular mechanisms lay at the center of this phenotypic resistance to therapy [14].

Lucas et al. observed that modification of the capsid protein hexon of adenovirus increased the transduction and cytotoxicity of pancreatic tumors [37]. These findings indicate a close association among cell survival mechanisms that regulate virus binding and entry to tumor cells, and thus, these can be exploited to improve therapeutic efficacy.

Viral transcription–translation: After successful entry in the host cell, the next challenge of the virus is productive transcription and translation of its genome, the production of viral progeny, and oncolysis. Therefore, the regulation of host transcription and translational machinery is another crucial step. However, tumor cells can regulate molecular processes and shut down virus transcription via epigenetic modification of either the host genome or the viral genome and by the expression of antiviral proteins such as the apolipoprotein B mRNA-editing enzyme (APOBEC).

For example, Shulak and colleagues demonstrated the role of tumor cell epigenetic modifications in the promotion of resistance against virotherapy. They observed that treatment with histone deacetylase inhibitors promoted VSV-mediated tumor cell death in a nuclear factor kappa-B (NF-kB) autophagy-dependent manner [38]. This study was in accordance with an earlier observation made by Bieler et al., showing that adenovirus oncolytic activity was promoted against glioblastoma cell lines [39]. Another confirmation came when it was observed that NF-κB inhibition could reverse the resistance phenotype, promoting the oncolytic potential of virotherapy [26]. Alternatively, epigenetic modifications in viruses can play a role as they can aid in circumventing the resistance developed by tumor cells. For example, it was observed that Zika virus with a higher frequency of genomic CpG dinucleotides exhibited a better oncolytic activity in glioblastoma cell lines [40].

Huff et al. demonstrated that APOBEC3 upregulation in an IFN-β dependent manner by tumor cells is an innate mechanism of resistance to RNA viral infection and worsens treatment outcome [41,42]. This suggests that APOBEC enzymes, via their mRNA editing nature, can have an important role in dictating the outcome of any nucleic-acid-based therapeutic or viral therapy. Similar mechanisms that are sensitive to nonself RNA molecules were observed against alphaviruses. Here, the activation of double-stranded-RNA-sensitive protein kinases in tumor cells led to restricted translation and increased sensitivity to interferon responses [43]. This limited viral replication in tumor cells exhibits a decreased interferon response.

Cell survival: Several survival proteins and their respective pathways play an important role in protecting tumor cells against viral infections and virus-induced cell death. Most of the studies (25 studies) retrieved from the systematic analysis found various molecular factors that play an important role in resistance towards oncolytic virotherapy by regulating cell survival. In addition to epigenetic modifications, promoting cell survival, regulation of autophagy, and apoptosis in tumor cells were found to promote resistance to virotherapy. For example, a study by Lv and colleagues demonstrated that vaccinia virus expressing IL-24 resulted in caspase-dependent apoptosis and decreased expression of the signal transducer and activator of transcription-3 (STAT3). In vivo, the modified virus also promoted apoptosis in lung cancer that was otherwise resistant to virus-induced cell death [44]. A similar study by Li et al. showed an increased oncolytic activity of virotherapy where tumor cell apoptosis was promoted by Beclin-1 activity expressed by adenoviruses [45]. A study by Colunga et al. indicated that loss of the autophagy protein sequestosome-1 (p62/SQSTM1) via calpain-dependent mechanisms can promote oncolytic activity in melanoma [46]. A role of autophagy in regulating oncolysis was supported by the observation that pharmacological regulation of autophagy improved Newcastle disease virus-mediated oncolysis in drug-resistant lung cancer cell lines [47]. Furthermore, several studies demonstrated that master regulators such as tumor protein p53 (TP53) and its activation abrogate viral infectivity and replication in tumors; however, this is limited to nonmalignant cells [48] and tumor cells that do not harbor TP53 mutations [48,49]. Various other cell survival regulators such as the inhibitor of nuclear factor kappa-B kinase subunit-beta (IKK) [50], inositol polyphosphate 5-phosphatase (Inpp5e) [51], mitogen-activated protein kinase (MAPK) [52], multidrug-resistance protein-1 (MDR1) [22], mitogen-activated protein kinase kinase (MEK) [53], second mitochondria-derived activator of caspases (SMAC) [54,55], Sirtuin 1 (SIRT1) [56], and N-myc proto-oncogene protein (MYCN) [57] have also been found associated with resistance to viral infection and replication in tumor cells.

Cell signaling: Intercellular communication between tumor cells has been well studied in the context of tumor progression and resistance to various immunotherapies, where tumor cells have been found to communicate and influence neighboring tumor cells via secreting cytokines, growth factors, metabolites, and extracellular vesicles to promote a protumoral environment [58]. Soluble signals released by infected cells can protect surrounding cells from viral infection, e.g., interferon responses [28,29,59,60,61,62,63,64,65,66,67]. Secretion of interferons by tumor cells infected with oncolytic viruses and the subsequent activation of the interferon-receptor (IFNR)-based Janus-kinase (JAK-STAT) pathway in surrounding tumor cells has been demonstrated; thus, also in this context, this pathway is an important mode of antiviral resistance [68]. Activation of interferon signaling has been observed to induce the expression of various antiviral proteins such as interferon-induced transmembrane protein 1 (IFITM1), interferon-induced protein with tetratricopeptide repeats (IFIT3), IFN-α inducible gene 6 (IFI6), 2′-5′-oligoadenylate synthetase 2 (OAS2), adenosine deaminases acting on RNA (ADAR), interferon-β (IFNβ), and tripartite motif containing 25 (TRIM25), which play a role in attenuating viral replication and the oncolysis of tumor cells [69]. These studies are supported by the observation that inhibitors of interferon signaling such as ruxolitinib can enhance the oncolytic potential of virotherapy in both in vitro and in vivo models [15,20,70,71].

Alternatively, the expression of the estrogen receptor on palbociclib-resistant breast cancer cells has been described to sensitize them towards adenoviral replication and oncolysis due to their low antiviral IFN response and increased cyclin-dependent kinase-2 activation [72].

Stromal cell responses: Although stromal cells make up an essential part of the tumor microenvironment, only eight studies explored them in relation to resistance against oncolytic virotherapy. For example, cancer-associated fibroblasts are often abundantly present in solid tumors. Unlike tumor cells, these cells do not have a compromised interferon signaling pathway and are not as permissive to viral infection [2]. Therefore, antiviral resistance demonstrated by these cells may affect the therapeutic efficiency of oncolytic virotherapy. Furthermore, Arwert et al. demonstrated that cancer-associated fibroblasts, via stimulator of interferon genes (STING) and interferon regulatory transcription factor 3 (IRF3), can influence neighboring cells and enable sensing of genomic stress in cancer cells to prevent infection [7]. Alternatively, in a secretome analysis study, fibroblasts were found to secrete protumoral growth factors during virotherapy that impeded viral replication in cancer cells [8]. Similarly, tumor-associated macrophages have been reported to induce a protective antiviral state via secretion of IFN-β in ovarian and breast tumors, rendering them resistant to oncolytic virotherapy [12]. This suggests that the regulatory role of such stromal cells requires more frequent and detailed observation in the context of preventing effective virotherapy.

Antiviral immune responses: Antiviral immune responses are generated during oncolytic virotherapy. For example, oncolytic virotherapy has been found to induce local immune responses and convert an immunologically “cold” tumor into a “hot”, immune-cell-infiltrated, tumor via the release of inflammatory signals post-viral oncolysis [73]. Here, interferon-based signaling is a major component of such inflammatory signals and plays an important role in the transformation of “cold-to-hot” tumors [73]. Although studies have demonstrated that blocking interferon responses improves viral infectivity in tumor cells, this may also limit the induction of interferon-dependent antitumor immune responses [33,68]. For example, Melero et al. demonstrated that intratumoral injections of Semliki forest virus (WT or replicon particles) depends on virus-induced interferon responses that enhance CD8+ T-cell-mediated antitumor activity, while furthermore, increased interferon signaling leads to a better survival [74]. In noncancer settings as well, interferon responses have been found to influence T-cell maturation and clonal development, where suppressing interferon signaling post-viral infection impedes antigen-specific T-cell development and activity [75]. Apart from interferon-based cellular immunity to viral infection, adaptive humoral and cellular responses mediated via lymphocytes also interfere with viral infection in the tumor.

Antibody responses can neutralize therapeutic viruses posing a challenge for multiple-dose regimens. Studies suggest that the use of a higher virus dosage for booster injections could overcome this limitation [4]. Alternatively, Biswas et al., using Newcastle disease virus, showed that complement-mediated opsonization was evaded when regulators of complement activating factors were incorporated in the viral envelope [76]. Zemp et al. reported that resistance to oncolytic myxoma virus in a glioma models involves a multifaceted cellular immune response that can be reversed with cyclophosphamide-mediated lymphoablation [77]. Similarly, cytolysis of virus-infected tumor cells by T-cells or NK cells has been observed to prevent the multiplication of viruses in the tumor microenvironment, decreasing the infection of surrounding tumor cells [78]. Tumor-associated macrophages may also prevent the infection of tumor cells by engulfing a large proportion of virus particles.

Systemic responses: Certain mechanisms that impede virotherapy occur at complex levels and exist due to the multidimensional interaction of various cells present in the microenvironment. For example, epithelial cells have been observed to act as a physical barrier and regulate the spatiotemporal spread of viruses in the tumor and periphery to target and lyse cancer cells [9,10]. Furthermore, angiogenesis and the vascular leakiness at local tumor sites have been proven to be a regulatory factor in viral spread and infection [11,79]. Limited viral replication in hypoxic zones of tumors is also responsible for resistance against virotherapy and requires engineering of a hypoxia-inducible expression system to restore oncolytic activity [80]. Alternatively, the presence of a dense extracellular matrix seems a limiting factor for spreading of viruses in tumors as well [32] and indicates the possibility of the inhibition of viral replication at a stage beyond viral entry [19,81]. Other than physical constraints, soluble signals such as prostaglandin [24] and vascular endothelial growth factor (VEGF) [11] have been observed to promote the accumulation of granulocytic myeloid suppressor cells correlating with resistance in the tumor microenvironment. Finally, healthy erythrocytes were recently discovered to play a role in decreasing viral spread in the body [82], indicating that certain mechanisms could be overlooked due to the choice of the model or by limiting our focus on disease pathology and not on the healthy state.

Thus, to successfully infect cancer cells, viruses need to cross a multitude of obstacles that arise at a systemic level. Challenges in the design of novel viral-vector-based therapeutics are therefore related to inefficient bio-distribution, suboptimal infection due to tumor hypoxia, overcoming vascular barriers, escaping antiviral immune responses, and intracellular antiviral molecular mechanisms. Moreover, it should be noted that such mechanisms when considered together might confer a certain degree of resistance to viral infection and subsequent oncolysis. However, due to the limited number of studies, the importance of the contribution of the different resistance mechanisms remains difficult to estimate.

Alternatively, multiple mechanisms in the tumor microenvironment have also been observed to promote virotherapy, which can be exploited to improve therapeutic efficacy. For example, an anti-inflammatory crosstalk between tumor cells and tumor-associated fibroblasts mediated by transforming growth factor-β (TGF-β) and fibroblast growth factor 2 (FGF2), respectively, has been found to sensitize both tumor cells and fibroblasts for viral infection [83]. Moreover, reovirus internalized by tumor-associated dendritic cells has been reported to promote virotherapy, providing protection against antibody-mediated neutralization [84,85]. Similarly, myeloid-derived suppressor cells have been found to act as a vehicle for tumor-specific delivery and targeting of oncolytic viruses [86]. Apart from viral delivery and protection, the role of myeloid cells has been studied extensively in the context of tumor antigen presentation [87] and antitumor immune responses post-virotherapy [88]. Therefore, exploiting mechanisms that allow us to sensitize tumor cells for viral infection and utilize stromal cells for viral delivery and tumor-associated myeloid cells for antitumor immunity could be some of the primary objectives for future research in the field of cancer virotherapy.

## 5. Conclusions

Through a systematic analysis of published literature, we identified key resistance mechanisms and cell types that are involved in undermining the therapeutic efficacy of oncolytic viruses. Importantly, our analysis demonstrated that resistance mechanisms can be classified as tumor-cell-mediated responses, stromal cell responses, antiviral immune responses, and systemic responses, which are involved in conferring resistance to various virus types in different cancers. Our results indicate the need to further explore known mechanisms such as interferon-mediated resistance and find novel factors such as epigenetic modifications, tumor heterogeneity, hypoxia, secreted factors, etc., that are responsible for promoting resistance to virotherapy. Of note, the analysis revealed the need to extensively assess the role of stromal cells such as fibroblasts, epithelial cells, endothelial cells, and tumor-associated myeloid cells, as to how they protect cancer cells from viral infection and oncolysis.

An extensive knowledge of such resistance mechanisms will aid researchers and clinicians in the rational design of virotherapy and for the implementation of appropriate therapeutic strategies. Research to date has shown the importance of acknowledging cancer as an evolving disease and the need to consider strategic therapeutics to combat resistance. Cell-based vector delivery to escape neutralizing antibodies, synthetic transgene expression circuits for efficient viral replication, suppression of antiviral interferon signaling, and directed evolution of viral vectors are a few of the existing techniques that can allow us to engineer and screen for viral vectors that can overcome their previous limitations. Thus, through our analysis and results, we encourage studies that explore resistance towards oncolytic viruses and hope to inspire the rational design of oncolytic viruses that can overcome therapeutic resistance.

## Data Availability

Attached as Supplementary Materials.

## Supplementary Materials

The following are available online at https://www.mdpi.com/article/10.3390/vaccines9101166/s1, Supplementary Information File S1 (.pdf), and Table S1: List of articles included in the systematic analysis, with the information of the first authors’ name, PUBMED identity number, DOI number, year of publication, cancer type, viral vector platforms, resistance mechanisms, and source of resistance to virotherapy. Table S1 is attached as a .xlsx file.
