# Supplementary material for: Resistance Mechanisms Influencing Oncolytic Virotherapy, a Systematic Analysis
Source: Vaccines (Basel). 2021 Oct 12;9(10):1166. doi: 10.3390/vaccines9101166 (PMC8537623; doi:10.3390/vaccines9101166)
Supplement: Supplementary file 1 [file vaccines-09-01166-s001.zip › supplementary material.pdf]

Supplementary information

- Search strategy used to retrieve articles from PUBMED:
  "resistance"[tiab] AND "oncolytic"[tiab]

- Supplementary table 1: List of articles included in the systematic analysis, with the information of first authors' name, PUBMED identity-number, doi-number, year of publication, cancer-type, viral vector platforms, resistance mechanisms and source of resistance to virotherapy. *Attached as an excel file.*
